# Sex Disparities in Bladder Cancer Diagnosis and Treatment

**DOI:** 10.3390/cancers16234100

**Published:** 2024-12-07

**Authors:** Géraldine Pignot, Philippe Barthélémy, Delphine Borchiellini

**Affiliations:** 1 Department of Surgical Oncology 2, Institut Paoli-Calmettes, 13009 Marseille, France; 2 Medical Oncology Unit, ICANS, Hôpitaux Universitaires de Strasbourg, 67200 Strasbourg, France; p.barthelemy@icans.eu; 3 Medical Oncology Unit, Centre Antoine Lacassagne, Université Côte d’Azur, 06000 Nice, France; delphine.borchiellini@nice.unicancer.fr

**Keywords:** bladder cancer, sex differences, immunotherapy, prognosis, treatment

## Abstract

Gender differences in prevalence, tumor invasiveness, response to treatment, and clinical outcomes exist in different types of cancer. The aim of this article is to summarize the sex disparities in bladder cancer diagnosis and treatment and try to suggest areas for improvement. Although men are at a higher risk of developing bladder tumors, women tend to be diagnosed with more advanced stages at diagnosis and are more likely to present with upfront muscle-invasive disease. Non-urothelial histological subtypes are more frequently reported in women. Regarding non-muscle-invasive bladder cancer (NMIBC), several studies have shown that women have a higher risk of disease recurrence after treatment with Bacillus Calmette–Guerin, due to different immunogenicities. In localized muscle-invasive bladder cancer (MIBC), neoadjuvant chemotherapy and cystectomy are less likely to be performed on women and sexual-sparing procedures with neobladder diversion are rarely offered. Finally, women appear to have a poorer prognosis than men, potentially due to the sex-associated intrinsic features of hosts and tumors that may drive differential therapeutic responses, particularly to immune-based therapies. Women are also more likely to develop severe adverse events related to systemic therapies and are underrepresented in randomized studies, leading to a gap between the real world and trials. In conclusion, studies investigating the role of sex and gender are urgently needed to improve the management of urothelial carcinoma.

## Simple Summary

The prevalence and tumor aggressiveness of urothelial carcinoma differ between men and women. This article summarizes the gender differences, especially regarding prognosis and response to treatment, as well as surgical considerations around cystectomy, and highlights the need to personalize the management of bladder cancer by taking these disparities into account.

## 1. Introduction

A wide disparity between men and women has been reported in terms of the cancer incidence, tumor aggressiveness, prognosis, and treatment response of different types of cancer [1]. However, only limited data are available to better understand the molecular basis underlying these sex disparities. Several explanations could be the sex-associated intrinsic features of hosts and tumors, including hormonal status, an enriched tumor microenvironment, a lower tumor mutational burden, and other epigenetic differences such as sex-specific DNA methylation and chromatin conformations [1,2]. Despite the acknowledged biological and physiological differences between men and women, little is known about the effect of patients’ sex on the efficacy and safety of systemic treatment, especially immune checkpoint inhibitors. The literature on the potential role played by sex in influencing drug pharmacokinetics and pharmacodynamics is poor and the efficacy of new treatments are rarely evaluated taking sex into account. Moreover, the sex ratio in several cancers favors men, and women represent only a very small sample in clinical trials, leading to discrepancies between phase III trials results and real-world data [3]. Although the importance of gender and sex inclusion in oncology practice is increasingly recognized, its application remains limited [4,5].

In bladder cancer, the recent implementation of immune checkpoint inhibitors and antibody–drug conjugates have changed the cancer treatment landscape in metastatic urothelial carcinoma treatment during the past decade [6,7,8]. Recent trials have also changed the paradigm in perioperative settings [9,10]. However, whether the efficacy and safety of such treatment are similar in women and men remains questionable [11]. Surgical management is also rapidly evolving but with relevant sex-disparities, regarding sex-sparing procedures or urinary diversion [12,13].

The aim of this article is to summarize the sex disparities in bladder cancer diagnosis and treatment, to highlight barriers for equity, and to try to suggest areas for improvement.

## 2. Methods

A scoping review was conducted to present the latest trends in sex/gender analysis in bladder cancer, with the aim of identifying knowledge gaps and investigating research conduct.

The protocol was reported following the PRISMA extension for scoping reviews (PRISMA-ScR). The peer-reviewed scientific literature was searched in PubMed, Google Scholar, and Scopus with keywords for cancer, bladder, urothelial, sex, and gender. The eligibility criterion consisted of human studies published between January 2005 and August 2024 in English. Screening, data extraction, data analysis, and critical quality appraisal of all texts were conducted. Of a total of 1287 initial hits, 378 duplicates were removed and 612 hits were excluded because they were out of our scope. After classifying by cancer type, and after full-text articles were assessed for eligibility, a total of 47 studies dealing with urothelial carcinoma were ultimately included in qualitative synthesis. The types of eligible studies included basic research studies, clinical studies, meta-analyses, and reviews. The selected articles were used to provide a comprehensive report on the state of sex/gender findings in bladder cancer.

**Figure 1:**
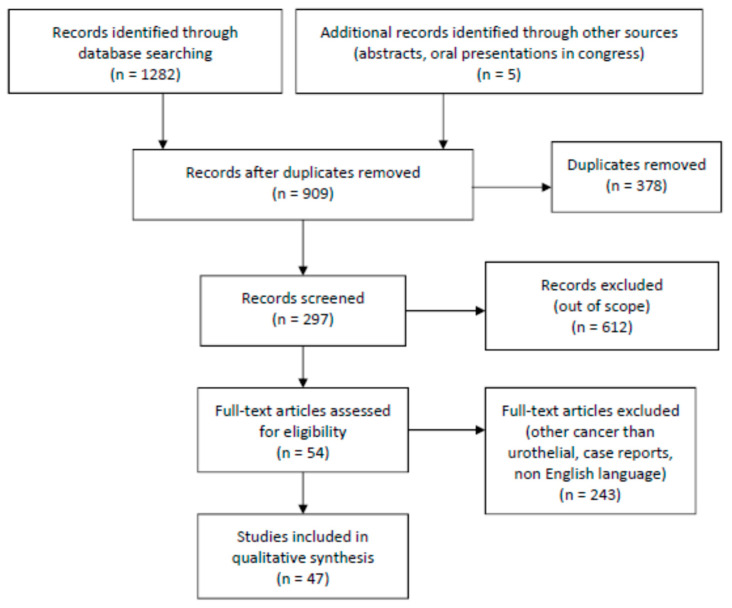
Flowchart diagram (PRISMA).

A flow diagram of the source selection and screening process is presented in Figure 1.

## 3. Gender Differences in Bladder Cancer Diagnosis

Bladder cancer is five times more common in men than in women, mainly due to tobacco and environmental carcinogen exposure [14]. This sex-associated difference in urothelial carcinoma incidence could also be influenced by sex hormones and tumor genetics. Sex hormones and their corresponding receptors may play a role in the occurrence and development of bladder cancer, and the presence of the androgen receptor (AR) gene, which is situated on the X chromosome, could potentially explain the differences in bladder cancer occurrence across genders [15].

Horstmann et al. found that women are older at the age of the detection of bladder cancer [16]. In a prospective cohort, postmenopause and early menopause are associated with an increased risk of bladder cancer [17].

Although men have a higher risk of developing a bladder tumor, women tend to be diagnosed more frequently, with >6-month delay, and to present with a more advanced stage at diagnosis [18,19]. This could be due to different symptoms at presentation, especially a lower frequency of macroscopic hematuria and higher occurrence of urinary tract irritative symptoms, leading to overlooked bladder tumors and an increased rate of the development of metastatic disease [19].

In a recent population-based analytic study assessing sex differences in muscle-invasive bladder tumors using data from a cancer national registry, a history of non-muscle-invasive bladder cancer was less frequent in women than men (8.9% versus 26%) [13]. Similarly, Scheller et al. found that females were more likely to present with muscle-invasive bladder cancer (MIBC) upon a first diagnosis [20].

In the TNM 2017 classification, there are no differences in terms of the staging of bladder cancer between men and women. The T4a stage is defined as the involvement of the prostate, seminal vesicles, vagina, or uterus. There are no data confirming the similar prognosis of this T4a stage in women (uterus, vagina) compared to men (prostate, seminal vesicles).

Moreover, the non-urothelial histological subtype is more frequently reported in women [13]. This is consistent with recent molecular data suggesting that female tumors exhibit an enrichment of the basal subtype compared to their male counterparts [21]. These histological differences could be partially explained by sex hormones but also by the bladder microbiome and chronic urinary tract infection (defined as an infection of the urinary tract that either does not respond to treatment or keeps recurring) [22]. Persistent exposure to chronic infection-related inflammation is associated with an increased risk of cancer [23]. On another hand, chronic inflammatory disease could induce squamous dedifferentiation. In the series of Poli et al., squamous cell carcinoma was four times more common in women than in men (12.9% versus 3.0%) while there was no significant difference regarding in the frequency of neuroendocrine carcinoma (2.4% versus 2.5%) [13]. These results are in agreement with a recent SEER database analysis, showing a higher frequency of squamous cell carcinoma in females, while adenocarcinoma, neuroendocrine tumors, and other histological variants were less frequent [24].

## 4. Gender Differences in NMIBC Management

The trans-urethral resection of a bladder tumor (TURBT) is the first step of the management of non-muscle-invasive bladder cancer (NMIBC). This surgical procedure is very important from a diagnostic and treatment point of view.

One of the major complications of the TURBT is bladder perforation, with a mean incidence of 2.4% and a risk of extravesical tumor seeding, potentially leading to an increased risk of metastasis and death [25]. Regarding the risk factors for bladder perforation during the TURBT, women may have a greater risk for perforation due to a thinner bladder wall [26]. On the contrary, the lack of a prostate and prostatic urethra may probably decrease the risk of the lymphovascular extension of tumors [27].

In NMIBC, women are also more likely to have a higher stage at presentation, while a higher prevalence of CIS is observed in males [20,28]. Several studies regarding gender differences have shown that females have a greater disease recurrence and worse outcomes [29,30]. A recent meta-analysis comprising 23,754 patients confirmed that women were at an increased risk of disease recurrence after the local treatment of NMIBC compared to men (hazard ratio [HR] = 1.11; 95% CI: 1.01–1.23; *p* = 0.03), especially after treatment with Bacillus Calmette–Guerin (BCG; HR = 1.64; 95% CI: 1.13–2.39; *p* = 0.01) [29]. These data suggest the reduced effectiveness of BCG treatment in women. Indeed, sex differences are prominent in response to Bacillus Calmette–Guerin immunotherapy used in the treatment of non-muscle-invasive bladder cancer, mainly due to different local bladder-resident immune populations. Women seem to experience a lower immune response after treatment, increasing their risk of being BCG-unresponsive [21].

Surprisingly, the progression of low-risk NMIBC is also more common in women [20]. This suggests a specific pathological pattern, perhaps associated with FGFR gene mutation, which could confer a risk of progression in these theoretically low-risk tumors.

## 5. Gender Differences in Localized MIBC Management

If women are more likely to be diagnosed at a more advanced stage, often muscle-invasive, their management seems to be quite different compared to that of men. Palliative treatment is more frequently proposed (46.8% versus 26.6%), not only for metastatic disease but also for patients with localized disease [13]. Indeed, cystectomy is less likely to be performed on women (45.1% versus 62.7%), as well as neoadjuvant chemotherapy (12.9% versus 19.2%), despite the fact that it is the optimal treatment option that confers the best survival rate in this setting.

It is not easy to explain this difference in terms of clinical management. Two hypotheses can be put forward, the first being that urologists are less accustomed to performing cystectomy on women and less familiar with surgical techniques, especially conservative ones. The second is that women refuse this surgery because of the potential impact on body image (notably in case of external derivation), on sexual activity, and on quality of life. Indeed, radical cystectomy is a major intervention with morbidity rates that should not be disregarded, with serious psychological and social drawbacks. Over the past decade, sex-sparing techniques are gaining popularity, with the aim of achieving definitive oncological control while attempting to preserve sexual function [31,32].

However, a well-documented gender bias exists in the assessment of sexual outcomes for women undergoing cystectomy. Many women report receiving inadequate preoperative counseling regarding the risks of sexual dysfunction, nerve-sparing techniques, and post-operative sexual health, regardless of the disease stage or receipt of chemotherapy [33]. The main reasons for this gender disparity have been documented to include an advanced patient age, inadequate time, and the uncertainty of baseline function [34,35].

In female patients, the standard surgical procedure is represented by anterior pelvic exenteration involving the en bloc resection of the bladder and adjacent pelvic organs, including the uterus, ovaries, anterior vaginal wall, and, in many cases, urethra. However, sexual dysfunction being derived from such a highly demolitive surgery is a key concern [36]. Functional outcomes among women undergoing radical cystectomy are understudied, with limitations stemming from the use of validated questionnaires, heterogeneous patient populations, and small sample sizes [37,38]. Despite the high risk of sexual dysfunction after cystectomy, there are little data and attention given to these issues in women, which contrasts with the level of attention paid to the sexual function of men undergoing similar urologic procedures.

The consequences of anterior exenteration are numerous. Patients may experience the following:

- Neurovascular bundle damage, since the pelvic sensory fibers of the inferior hypogastric plexus are mainly concentrated in the posterior fornix along the lateral sides of the cervix and rectum and are therefore destroyed by exenteration. Autonomic and nociceptive nerve injuries are often associated with pain disorders, such as dyspareunia, vulvodynia, and vaginismus [31].
- The shortening or narrowing of the vagina.
- The frequent devascularization and denervation of the clitoris in the case of urethra removal.
- Voiding issues when performing with an orthotopic neobladder [39,40].

Recently, gender differences in oncological and functional outcomes after radical cystectomy have received increased attention. According to current guidelines, sexual-sparing cystectomy is an option to consider for women highly motivated to preserve sexual function as soon as the following strict oncological inclusion criteria are met: a localized tumor (cT2) detected in preoperative imaging away from the bladder neck, trigone, or dorsolateral bladder walls [6]. In these well-selected women, organ-sparing approaches can be performed safely without negatively impacting oncological outcomes [41].

Vaginal preservation is important since dyspareunia is the most common post-operative change in sexual function after radical cystectomy [42]. Multiparametric bladder MRI is the best imaging to confirm the absence of the involvement of the trigone or the dorsolateral bladder walls, allowing for the full preservation of the anterior vaginal wall and reducing the risk of damage to neurovascular paraurethral structures, which is crucial for both sexual and continence functionality.

Uterus-sparing surgery would bring noticeable progression in sexual outcomes. Moreover, in the case of orthotopic bladder substitution, the continence rate has been shown to be significantly higher and the clean intermittent catheterization significantly lower compared to standard radical cystectomy [43,44].

Regarding the low incidence of ovarian cancer, it does not seem logical to perform systematic concomitant oophorectomy during radical cystectomy in a patient with no history of hereditary breast or ovarian cancer [45,46]. Moreover, oophorectomy-induced surgical menopause has been shown to increase the risk of osteoporosis, cognitive impairment, cardiovascular disease, and all-cause mortality and is associated with poorer QOL scores compared to natural menopause [47,48,49].

Based on these considerations, anterior exenteration “d’office” is no longer acceptable. For well-selected sexually active patients, sex-sparing cystectomy is oncologically safe and may offer functional benefits in preserving pelvic reproductive organs and their nerve structures, with a significant impact on QOL both in terms of sexual health and urinary continence.

Another gender difference is about urinary diversion. While urinary functional outcomes are good, the neobladder is rarely proposed to women, mainly due to the heterogeneity of practices regarding adherence to guidelines and the expertise of centers [12,13].

## 6. Prognosis

Women appear to have a poorer prognosis than men with a higher overall mortality rate, with female gender being classically found to be an independent risk factor for mortality [13,50]. A meta-analysis including 27912 patients showed that female sex is associated with a poorer prognosis (HR: 1.20; 95% CI: 1.09–1.32) after cystectomy [51]. A 2015 Austrian study including 27773 patients confirmed this sex-related difference, with a specific survival identical in the pT1 subgroup but less favorable for women in the MIBC group [52]. A population study using MarketScan databases suggested that the difference in survival may be related to the longer time taken to diagnose women due to more frequent differential diagnoses [19]. The impact of hormonal features on treatment response was also discussed.

## 7. Gender Differences in Response to Systemic Therapies

Immune checkpoint inhibitors and antibody–drug conjugates have changed the therapeutic landscape of urothelial carcinoma treatment in recent years. Several sex-specific differences have been identified in terms of the efficacy of systemic treatment, as monotherapy or in combination, in both metastatic and adjuvant settings, although data remain controversial. It is commonly accepted that the sex-associated intrinsic features of hosts and tumors may potentially drive differential disease progression and a therapeutic response in urothelial carcinoma of the bladder. Regarding immunotherapy, emerging findings from recent trials are suggestive of differential outcomes in females compared to males. Sex-specific differences in the way the immune system functions and responds to pathogenic insults are well established [21]. Women appear to have an enriched microenvironment for certain immune cell types, particularly antiviral T cells, with different innate and adaptive responses to men [53,54].

While male patients typically have hot tumors, which are clearly visible to the immune system, females often have cold tumors, indistinguishable by the immune system, and therefore respond poorly to immunotherapy [55,56]. While some specific immunological differences between females and males are described throughout life, some discrepancies may appear during hormonal variations, especially after puberty and before menopause, suggesting that they may be likely driven by sex hormones [53,57].

Regarding tumor mutational burden (TMB), several studies have suggested that it is generally lower in female than in male tumors, especially in bladder cancer but also in kidney, liver, and skin cancers [58]. Fewer immunogenic neoantigens are described in tumors with low tumor mutational burden (TMB) [59]. There is also a difference in mutational burden potentially linked to a decrease in the expression of DNA mismatch repair genes. Thus, in female patients, there is a lower antigenicity of tumors which could lead to a less effective antitumor immune response to treatment. Other epigenetic differences that may contribute to the poor response to immunotherapies include DNA methylation and chromatin conformations which appear to differ between men and women [1,2,58]. Finally, behavioral and social factors may also play a role in the gender disparity in response to immunotherapy. In addition to smoking and alcohol consumption, women are also more exposed to mental loads, emotional factors, and violence [60]. Posttraumatic stress may affect gonadal steroid secretion in a sex- and hormone-dependent fashion [61].

There is an extensive literature on the potential role played by sex in influencing drug pharmacokinetics, pharmacodynamics, and efficacy. However, new therapeutic approaches are rarely tested taking sex into account, and data from clinical trials are controversial. In a systemic review and meta-analysis from 20 randomized controlled trials of immune checkpoints inhibitors (ipilimumab, tremelimumab, nivolumab, or pembrolizumab), Conforti et al. reported a significant difference in efficacy between men and women in terms of overall survival (HR = 0.72 (95% CI 0.65–0.79) in male patients versus HR = 0.86 (95% CI 0.79–0.93) in women; *p* = 0.0019) [62]. The results of this study showed that immune checkpoint inhibitors can improve overall survival for patients of both sexes but that men have a larger treatment effect from these drugs versus control treatments than women. It is noteworthy that various types of metastatic cancer were included in this analysis, the most common being melanoma (32%) and non-small-cell lung cancer (31%). In contrast, specifically regarding trials assessing immunotherapy-based combination therapy for metastatic urothelial carcinoma, a more recent systematic review and meta-analysis did not show statistically significant differences according to gender in first- or second-line treatment; however, regarding antibody–drug conjugates, males showed a significant survival advantage in second-line treatment for enfortumab vedotin [11]. Two recent phase III studies have recently changed the standard landscape of the first-line treatment of advanced/metastatic urothelial carcinoma by demonstrating an improvement in overall survival compared to standard chemotherapy. The CheckMate 901 trial demonstrated statistically significant and clinically meaningful improvements in overall survival (HR = 0.78 (0.63–0.96)) with nivolumab (NIVO) + gemcitabine–cisplatin (GC) versus GC alone as first-line treatment for unresectable or metastatic urothelial carcinoma [7]. Women represented 23% of the patients in this trial, and subgroup analysis showed that they had a smaller therapeutic effect than men (HR = 0.82 (95%CI 0.54–1.26) versus HR = 0.76 (95%CI 0.60–0.97)). The second trial, namely EV-302, also showed a significant improvement in overall survival with a still much better benefit (HR = 0.47 (95%CI 0.38–0.58)), with the risk of death being reduced by 53% in patients who received enfortumab vedotin + pembrolizumab compared to standard chemotherapy [8]. Women represented 23% of the patients, with no difference in terms of the treatment effect in the subgroup analysis of OS (HR = 0.51 (95%CI 0.32–0.80) versus HR = 0.47 (95%CI 0.36–0.60)).

In perioperative settings, data are also equivocal. A recent subgroup analysis from the CheckMate 274 phase III study assessing adjuvant nivolumab versus a placebo after cystectomy for MIBC of a high risk of recurrence (ypT2-4, or pT3-4, and/or (y)pN+) suggested that female patients did not benefit from this adjuvant strategy (HR = 0.95 [0.58–1.55]) compared to male patients (HR = 0.72 [0.56–0.93]) [9]. Even if the number of patients was lower (n = 169 women), the absence of a difference between the two arms was clear (34 deaths/88 in nivolumab arm and 33/81 in placebo arm) and should lead to us questioning ourselves on the potential hypotheses explaining such a result. More recently, results from the NIAGARA trial have been reported, showing a statistically significant and clinically meaningful improvement in overall survival (HR = 0.75 (95%CI 0.59–0.93); *p* = 0.01) with perioperative immunotherapy with durvalumab associated with neoadjuvant chemotherapy compared to neoadjuvant chemotherapy alone, supporting this strategy as a potential new standard treatment for patients with cisplatin-eligible MIBC [10]. Women represented only 18% of the patients included in this trial, but they seemed to benefit from this new perioperative strategy at least as much as the men (HR = 0.56 (95%CI 0.32–0.94) versus HR = 0.80 (95%CI 0.62–1.02)).

These discrepancies between studies highlight that care providers cannot extrapolate data from men and assume that they are applicable to women.

## 8. Gender Differences in Toxicities Related to Systemic Therapies

From a physiological point of view, even if a dose is corrected for weight and height, there are pharmacodynamic and pharmacokinetic differences in the adsorption, distribution, metabolism, and elimination of many medicines, which could help to explain sex-related differences in terms of the adverse events of chemotherapy or immunotherapy [29,63,64,65,66].

Sarcopenia is one of the putative explanations associated with chemotherapy dose-limiting toxicity in several cancers [67]. Menopause is associated with hormonal changes, which could accelerate or lead to sarcopenia [68,69,70]. Regarding bladder cancer, it is well known that 40 to 55% of women treated by radical cystectomy for a muscle-invasive tumor are sarcopenic (skeletal muscle index [SMI] at the third lumbar vertebrae <39 cm^2^/m^2^) [71,72]. Sarcopenia is a significant risk factor for postoperative complications and mortality after cystectomy, but it also appears to be an independent prognostic factor for renal impairment during neoadjuvant chemotherapy [71,72,73,74,75].

It is well known that immunotherapies have fewer side effects than chemotherapy, with a very different toxicity profile and specific adverse events. One of the future goals will be to be able to identify patients at the highest risk of treatment-related toxicity. In a recent post hoc analysis from SWOG phase II and III clinical trials conducted between 1980 and 2019, it appeared that the risk of cardiotoxicity was higher for women than for men [76]. Indeed, severe adverse events were reported by women in 73.9% of cases under chemotherapy (versus 67.6% for men) and in 56.6% under immunotherapy (versus 48.8% for men), which was statistically significant and clinically meaningful. This difference was also observed with targeted therapies (68.6% versus 62.2%). Moreover, women were more likely to develop severe and symptomatic toxicities (+30% under chemotherapy or targeted therapies and +66% under immunotherapy), suggesting that these specificities should be monitored and treated appropriately.

## 9. Inclusion of Women in Clinical Trials

Sex discrimination in medical trials is recognized as a major problem, leading to the under-representation of women in biomedical research [77,78]. Indeed, women comprise only 40% of the overall research population, with a wide variation by country and by cancer type (e.g., 13–100%) [79]. Bladder cancer has lower odds of adequate female representation when compared to all other cancer sites [80].

Several explanations could be discussed to explain this observation. This may be attributed to the biological, clinical, or social characteristics of the patients, the study design of the trial, or the physicians [3]. Firstly, eligibility criteria may impact the ability of women to participate in clinical trials. In addition to the usual exclusion criteria related to fertility and/or childbearing potential, hormonal status, or contraindicated medication, normative laboratory values (e.g., hemoglobin levels) and other measurements (e.g., BMI) included in eligibility criteria should be adjusted for sex and related demographic variables.

This is particularly true for surgical trials on surgical and perioperative treatment strategies since the cystectomy procedure is significantly different between women and men due various anatomical considerations that could interfere with the functional endpoints.

It is noteworthy that, if they meet eligibility criteria, there is no evidence that women are “less willing” to volunteer in clinical research. Given the opportunity to participate, women appear to sign up more often than their male counterparts. However, they are more willing to complete surveys and less willing to undergo tedious and time-consuming procedures [81]. Secondly, family commitments could interfere in the decision of female patients. Women are often the primary caregivers at home, and this could be an impactful barrier to accept signing up. The lack of flexibility and the high number of scheduled appointments, visits, and other research procedures is particularly prohibitive as women often serve dual roles as employees and as caregivers at home. Finally, yet importantly, physician-related bias play a major role in the decision to include patients in clinical trials. The longer time needed for communication and/or explanation, such as complex documentation leading to a longer time for the acquisition of informed consent, could disadvantage women [3].

The underrepresentation of women in randomized intervention studies leads to a large gap between real-world adults and those who participate in trials. This gap attempts to extrapolate the results from studies not representative of the real world. Even when women are included in trials, the results are often not stratified by sex, and the analysis of sex-based differences is uncommon [82]. Therefore, the risk of adverse events and the real effectiveness of these treatments is largely unknown in women. Physicians and scientists do not have adequate, evidence-based information to guide the care of women and may offer suboptimal treatment or alternatively expose them to unanticipated harm.

It is necessary to be able to provide clear information on the sex (male, female) and gender (male, female) of patients included in clinical trials to enable the appropriate stratification of patients [83]. Finally, increasing the number of women who are authors, lead investigators, and co-investigators has been demonstrated to correlate with the enrollment of female participants and a greater likelihood of reporting sex/gender specific analysis. [84]

Ultimately, several mechanisms leading to gender disparities may interfere, including medical bias and differential initial symptoms increasing the risk of overlooking bladder tumors in women, differential surgical management, with sex-sparing procedures rarely offered to women, poorer prognosis, and higher risk of recurrence after treatment, partly due to differential immunogenicity, as well as the underrepresentation of women in clinical trials.

## 10. Conclusions

There are gender differences in the prevalence, tumor invasiveness, response to treatment, and clinical outcomes of bladder cancer, suggesting that, although not yet integrated, the proper consideration of sex differences in clinical practice is needed to fill current gaps.

As personalized medicine is a growing area of interest, it will be crucial to identify predictive factors, with possible differences between men and women, to better understand and overcome resistance mechanisms, to predict responses to systemic treatment, and to select the most appropriate strategy for a given patient at the individual level.

This seems particularly relevant with the increasing use of immunotherapy in bladder cancer treatment, in metastatic disease, and in perioperative settings. Among the proposed intrinsic tumor/host factors that may influence responses to immune-based therapies, patients’ gender remains a challenging consideration that deserves further attention. Efforts should also be made to offer adequate surgical procedures to women, taking into account expectations in terms of continence and sexuality.

Studies investigating the role of sex and gender are urgently needed to improve the management of urothelial carcinoma and implement precision medicine, both medically and surgically, for patients with bladder cancer.
